# Drunk or Sober?

**Published:** 1895-08-24

**Authors:** 


### Drunk or Sober?

The varieties of drunkenness are well nigh infinite. When, then, we find the same agent in the one case rendering a man gently jovial, in another making him dead drunk, and in others producing every possible intermediate condition, and when we think of the wide differences which exist in temperament and in behaviour between men who, whatever their peculiarities, are well within the bounds of sobriety and reason, we need not hesitate to admit the difficulty of sometimes determining whether in a given case a man is drunk or sober. And yet the importance of this decision is immense. Not only character, but even life may, and very often does, depend upon the verdict whether an individual who shows some of the ordinary signs of drunkenness is really suffering from alcohol or is the victim of assault, or accident, or disease.

We do not here propose to enter into the points which decide the diagnosis, but the matter is one of such public importance, the difficulty is of such frequent occurrence, and, we may add, the problem is so often solved wrongly, that we think it well to draw attention to the very considerable number of conditions which may give rise to symptoms simulating drunkenness.

Roughly, we may say that the commonly-accepted signs by which the man in the street recognises the effects of alcohol are unsteady gait, incoherent speech, extravagant behaviour, and drowsy helplessness. Doubtless any one of these coming on suddenly in one who, up to the moment, had been as other men, would excite suspicion of disease. But the spectators do not see the beginning of the case. Till a man is helpless or obstreperous but little notice is taken of his vagaries, and in ninety-nine cases out of a hundred the decision must be arrived at from the actual condition visible to the eye, and a diagnosis is not always easy on such imperfect evidence.

The first and most obvious signs of drunkenness are those dependent on unsteadiness of gait. From the street boy's point of view, these are always productive of hilarity, and even the better instructed are apt to look on inability to walk straight as conclusive; and yet how many maladies produce the same condition. Many a man in the early stages of locomotor ataxy has lost his character from his tendency to stagger in the dark. Then those conditions which go by the name of Menière's disease may closely imitate the effects of drink. To the sufferer, when the paroxysm comes on, the sensation is of violent noises in the ear accompanied by a feeling of being whirled through space, or as if the road, the houses, and everything in sight, were flying in large circles round about him, and no wonder that he seizes the nearest lamp post or sinks down sick and helpless on the pavement, clutching for security anything within his reach. To the by-stander, however, he is a picture of the most abject drunkenness. But it is not only sufferers from organic diseases who suffer from vertigo. A very simple attack of indigestion may in some produce it, and many sufferers from migraine lose their balance. People sometimes also unknowingly take drugs which have the same effect. In both these cases speech also may be affected, and explanation may be difficult. Disorder of speech, however, is usually connected with more serious disease, and a man with a small hæmorrhage on his brain may stagger in speech as well as with his legs, and may be "run in" as drunk when his very life depends on proper treatment. The same may happen after injury of the skull. A man, full of drink, may in a street row receive a blow which causes fracture of the skull, but he may not drop. He may walk away far from any evidence of riot, and then sit down, and, becoming comatose, may be taken to the station as being dead drunk. And here, if there be no obvious wound, everything is against him. The smell of his breath condemns him to the police-cell instead of the hospital ward, and only at the post-mortem is it found that his skull is broken, and that the pressure on his brain might have been relieved. Such cases as this occur almost every month. But, in fact, in the diagnosis of drunkenness the possibilities of error are endless. Poison, uræmia, post-epileptic states, the excited stages of general paralysis, sudden outbreaks of mania, the occurrence of diabetic coma, the onset of acute febrile disease, and even mere exhaustion and fatigue may all produce symptoms simulating the effects of drink. We cannot then too strongly emphasize the necessity of medical examination whenever there can be the slightest doubt whether a man is drunk or sober, nor can we too strongly urge any medical man who is called to such a case to be wary in his dealings with it, to distrust first impressions, to enter into all the symptoms, however plain the case may seem, and to remember the endless pitfalls in the way of hasty diagnosis.

